# Electronic cigarette use in relation to changes in smoking status and respiratory symptoms

**DOI:** 10.18332/tid/176949

**Published:** 2024-01-22

**Authors:** Linnéa Hedman, Gustaf Lyytinen, Helena Backman, Magnus Lundbäck, Caroline Stridsman, Anne Lindberg, Hannu Kankaanranta, Lina Rönnebjerg, Eva Rönmark, Linda Ekerljung

**Affiliations:** 1 Department of Public Health and Clinical Medicine, The OLIN Unit, Umeå University, Umeå, Sweden; 2 Department of Clinical Sciences, Division of Cardiovascular Medicine, Karolinska Institutet, Danderyd University Hospital, Stockholm, Sweden; 3 Krefting Research Center, Institute of Medicine, Sahlgrenska Academy, University of Gothenburg, Gothenburg, Sweden; 4 Department of Respiratory Medicine, Seinäjoki Central Hospital, Seinäjoki, Finland; 5 Faculty of Medicine and Health Technology, Tampere University, Tampere, Finland

**Keywords:** airways, ENDS, epidemiology, prospective, quitting smoking

## Abstract

**INTRODUCTION:**

How e-cigarette use relates to changes in smoking status and respiratory symptoms in the population remains controversial. The aim was to study the association between e-cigarette use and, changes in smoking status and changes in respiratory symptoms.

**METHODS:**

A prospective, population-based study of random samples of the population (age 16–69 years) was performed within The Obstructive Lung Disease in Northern Sweden (OLIN) study and West Sweden Asthma Study (WSAS). A validated postal questionnaire containing identical questions was used in OLIN and WSAS at baseline in 2006–2008 and at follow-up in 2016. In total, 17325 participated on both occasions. Questions about respiratory symptoms and tobacco smoking were included in both surveys, while e-cigarette use was added in 2016.

**RESULTS:**

In 2016, 1.6% used e-cigarettes, and it was significantly more common in persistent tobacco smokers (10.6%), than in those who quit smoking (2.1%), started smoking (7.8%), or had relapsed into tobacco smoking at follow-up (6.4%) (p<0.001). Among current smokers at baseline, tobacco smoking cessation was less common in e-cigarette users than e-cigarette non-users (14.2% vs 47.6%, p<0.001) and there was no association with a reduction in the number of tobacco cigarettes smoked per day. Those who were persistent smokers reported increasing respiratory symptoms. In contrast, the symptoms decreased among those who quit tobacco smoking, but there was no significant difference in respiratory symptoms between quitters with and without e-cigarette use.

**CONCLUSIONS:**

E-cigarette use was associated with persistent tobacco smoking and reporting respiratory symptoms. We found no association between e-cigarette use and tobacco smoking cessation, reduction of number of tobacco cigarettes smoked per day or reduction of respiratory symptoms.

## INTRODUCTION

Electronic cigarettes (e-cigarettes) are marketed as an alternative product to tobacco cigarettes, nowadays common among all age groups but with a considerable appeal among younger individuals. A European survey estimated that 34% of participants aged 14–18 years had tried e-cigarettes at least once^1^. A common public perception is that e-cigarettes are less addictive and harmful than tobacco cigarettes^2^. However, the aerosol produced by e-cigarettes contains several compounds hazardous to human health, such as volatile organic compounds, fine particulate matter, and nicotine^3^. Furthermore, the nicotine level in the e-cigarette aerosol is similar to that of tobacco cigarettes, thus capable of causing addiction^4^.

Those who advocate e-cigarettes claim that by switching from tobacco cigarettes to e-cigarettes, the exposure to toxic compounds from cigarette smoke is decreased. Therefore, e-cigarettes could be considered a smoking cessation product, which a Cochrane Review supports^5^. However, increasing evidence has emerged showing that e-cigarette use affects human health detrimentally with toxicity, respiratory disease, inflammation, and adverse cardiovascular effects^6-9^.

Population-based studies have shown that e-cigarette use encourages dual use of both electronic and tobacco cigarettes rather than successful smoking cessation^2,10,11^. It is well established that quitting smoking improves long-term health and reduces respiratory symptoms in smokers^12^. Additionally, cross-sectional studies have found that dual users had more respiratory symptoms than individuals using only one of the products^10,13^. However, the impact of e-cigarette use and tobacco smoking on longitudinal changes in smoking status and respiratory symptoms in the population remains poorly studied and is a controversial subject^7,14,15^.

Therefore, this population-based study aimed to evaluate the association between e-cigarette use, changes in smoking status, and changes in respiratory symptoms. First, if we assume that e-cigarettes are used as a smoking cessation aid, we hypothesize that smokers with respiratory symptoms at baseline are more likely to initiate e-cigarette use than smokers with fewer symptoms (hypothesis 1). Second, we hypothesize that smokers who also use e-cigarettes have a higher prevalence of respiratory symptoms than smokers not using e-cigarettes (hypothesis 2). Third, because e-cigarettes may have a disadvantageous effect on respiratory health, we hypothesize that smokers who quit tobacco cigarettes and switch to e-cigarettes have less reduction of respiratory symptoms than smokers who quit tobacco cigarettes without switching to e-cigarettes (hypothesis 3).

## METHODS

### Setting

The study was based on surveys performed within the Obstructive Lung Disease in Northern Sweden (OLIN) studies and West Sweden Asthma Study (WSAS). OLIN includes the county of Norrbotten in the north of Sweden (area: 97242 km^2^; inhabitants per km^2^: 2.6), and WSAS includes Västra Götaland in the southwest of Sweden (area: 23800 km^2^; inhabitants per km^2^: 73.3). OLIN and WSAS have used the same methods and identical questionnaires, enabling pooling of data.

### Study sampling and procedure

At baseline in 2006, OLIN conducted a postal questionnaire survey in random samples of the adult population aged 20–69 years. Of the 13702 invited, 10414 participated (76%). Similarly, WSAS conducted a postal questionnaire survey 2008 in a random sample aged 16–69 years. Of the 30000 invited, 18087 participated (60%). In 2016, both the OLIN and WSAS cohorts were followed up, and after three reminders, 8424 participated in OLIN and 11699 in WSAS. The cohorts have been described in detail previously, including the questionnaires used and non-response analyses^16-20^. The current study sample consists of the 17325 individuals in OLIN and WSAS that participated at baseline in 2006–2008 and at the follow-up in 2016, and had complete data on tobacco smoking and e-cigarette use. The basic characteristics of the participants at baseline are presented in the Supplementary file Table E1.

The studies were approved by the Regional Ethical review boards in Umeå (Dnr: 2015-404-31) and Gothenburg (Dnr 052-16). All participants gave their written informed consent to participate in the study as they returned the postal questionnaire.

### Questionnaire

The same validated questionnaire was used by OLIN and WSAS^20^. Briefly, the questionnaire included questions about respiratory symptoms during the last 12 months, current and previous smoking status, sex, age, and occupation. A question about e-cigarette use was added to the 2016 questionnaire.

### Definitions

*Current smoker* – affirmative answer to the question: ‘Do you smoke?’.

*Former smoker* – affirmative answer to the question: ‘Have you been a smoker but have stopped smoking more than one year ago?’.

*Non-smoker* – negative answers to both of the questions: ‘Do you smoke?’ and ‘Have you been a smoker but stopped smoking more than one year ago?’, i.e. neither a current smoker nor a former smoker.

Changes in smoking status between baseline in 2006–2008 and at follow-up in 2016 were defined as follows:

*Never smoker* – non-smoker in both surveys. *Sustained former smoker* – former smoker in both surveys.

*Quitter* – current smoker at baseline and former smoker at follow-up.

*Starter* – non-smoker at baseline and current smoker at follow-up.

*Relapsed* – former smoker at baseline and current smoker at follow-up.

*Persistent smoker* – current smoker in both surveys.

*E-cigarette use* – responses ‘sometimes’ or ‘daily’ to the question: ‘Do you use e-cigarettes?’.

Respiratory symptoms were defined by affirmative answers to the following questions^20^:

*Longstanding cough* – ‘Have you had a longstanding cough during the last year?’.

*Sputum production* – ‘Do you usually have phlegm when coughing, or do you have phlegm in your chest which is difficult to bring up?’.

*Chronic productive cough* – Sputum production and ‘Do you bring up phlegm on most days during periods of at least three months?’ and ‘Have you had such periods during at least two successive years?’.

*Wheeze in the last 12 months* – ‘Have you at any time during the last 12 months had wheezing or whistling in your chest?’.

*Recurrent wheeze* – ‘Do you usually have wheezing, whistling or a noisy sound in your chest when breathing?’.

*Dyspnea* – ‘Do you get short of breath when you walk with other people of your own age on level ground at normal pace?’, thus corresponding to the Modified Medical Research Council dyspnea scale (mMRC) >2.

*Any respiratory symptoms* – An affirmative answer to any questions on respiratory symptoms.

Socioeconomic status was based on the longest-held occupation and categorized according to Statistics Sweden (Socioeconomic Index, SEI) into professionals and executives, self-employed, non-manual workers, manual workers in industry, manual workers in service, students, and unspecified.

### Statistical analyses

Analyses were performed using IBM SPSS statistics version 26 (IBM, Armonk, NY, USA) and GraphPad Prism 9.1.0 (GraphPad Software Inc., CA, US). Differences in proportions or means between groups were analyzed by the chi-squared test, t-test or ANOVA. A p-value <0.05 was considered statistically significant. For questions on respiratory symptoms, missing answers to individual questions (<3%) were regarded as negative responses. The distribution of respiratory symptoms are presented as proportions and number of symptoms. Changes in respiratory symptoms between baseline and follow-up are presented as the number of reported symptoms (range: 0–5, mean and standard deviation). Among those who were current smokers at baseline, the association between changes in smoking status and e-cigarette use and its relation to respiratory symptoms, was analyzed by ordinal regression with the number of respiratory symptoms as the dependent variable. Comparison of respiratory symptoms between non-smokers, recent quitters and persistent smokers with and without e-cigarette use, was analyzed by logistic regression analysis with any respiratory symptoms as the dependent variable. The regression analyses were adjusted for sex, age, socioeconomic status and number of symptoms at baseline, and the results are expressed as odds ratios (OR) with 95% confidence intervals (CI).

## RESULTS

### Tobacco smoking

**Figure 1:**
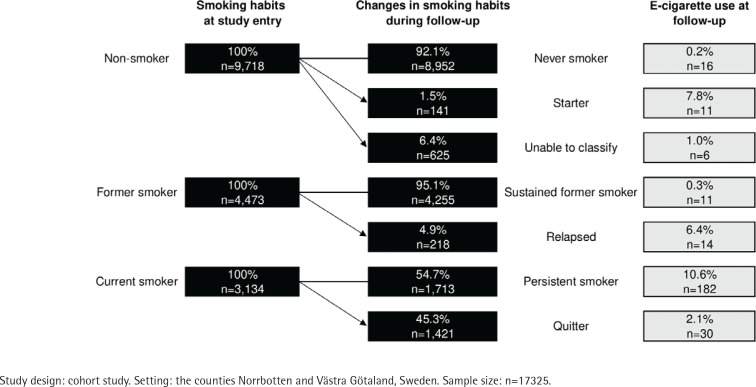
Tobacco smoking habits at baseline in 2006–2008, changes in smoking habits at follow-up, and e-cigarette use at follow-up in 2016, by tobacco smoking categories

Smoking status and cigarette consumption at baseline and at follow-up are presented in Table 1. At baseline in 2006–2008, 18.1% (n=3134) were current smokers. At follow-up in 2016, the prevalence of current smokers had decreased to 12.0%. Both at baseline and at follow-up, most current smokers reported that they smoked 5–14 cigarettes per day. Changes in smoking status are presented in Figure 1. Of the non-smokers at baseline, 92.1% (n=8952) remained never smokers, and 1.5% (n=141) had started smoking at follow-up. The changes in smoking status for the remaining 6.4% (n=625) could not be classified. Of the former smokers at baseline, 4.9% (n=218) had relapsed into current smoking at follow-up, and the rest were sustained former smokers. Of the current smokers at the baseline, 54.7% (n=1713) were persistent smokers, while 45.3% (n=1421) had quit smoking at follow-up, corresponding to an average annual quit rate of 5.0%.

**Table 1:** Smoking characteristics at baseline in 2006–2008 and at follow-up in 2016, and corresponding prevalence of e-cigarette use at follow-up^a^

*Smoking characteristics*	*Total*		*E-cigarette use*		
	*n/N*	*%*	*n/N*	*%*	*p^a^*
**Baseline**					
**Smoking status**					
Non-smoker	9718/17325	56.1	33/9718	0.3	
Former smoker	4473/17325	25.8	25/4473	0.6	
Current smoker	3134/17325	18.1	212/3134	6.8	<0.001
**Cigarettes per day among current smokers**					
<5	932/2970	31.4	47/932	5.0	
5–14	1378/2970	46.4	94/1378	6.8	
15–24	590/2970	19.9	57/590	9.7	
≥25	70/2970	2.4	11/70	15.7	<0.001
Missing	164				
**Follow-up**					
**Smoking status**					
Non-smoker	9469/17325	54.7	21/9469	0.2	
Former smoker	5784/17325	33.4	42/5784	0.7	
Current smoker	2072/17325	12.0	207/2072	10.0	<0.001
**Cigarettes per day among current smokers**					
<5	671/2008	33.4	55/671	8.2	
5–14	943/2008	47.0	87/943	9.2	
15–24	358/2008	17.8	53/358	14.8	
≥25	36/2008	1.8	6/36	16.7	0.003
Missing	64				

a Study design: cohort study. Setting: the counties Norrbotten and Västra Götaland, Sweden. Sample size: n=17325.

b χ^2^ test comparing proportion of e-cigarette use by smoking habits.

### E-cigarette use

The prevalence of e-cigarette use in 2016 was 1.6% (n=270), with similar estimates among men and women (1.4 and 1.7%, p=0.280). E-cigarette use was most common in the age group 20–29 years (2.3%), followed by 1.7% among those aged 40–49 years and 50–59 years, and 1.6% among those aged 16–19 years. Compared to non-smokers (0.3%) and former smokers (0.6%), e-cigarette use was significantly more common among those who were current smokers at baseline (6.8%, p<0.001), particularly among those who smoked more than 15 tobacco cigarettes per day (Table 1). Similar associations were seen in the cross-sectional analysis of the follow-up survey.

### Associations between e-cigarette use and changes in smoking status

**Figure 2:**
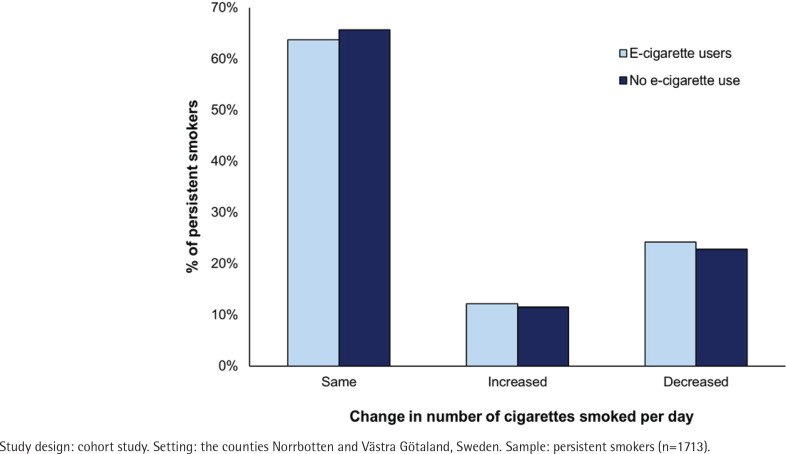
Change in number of cigarettes smoked per day from 2006–2008 to 2016 among persistent smokers, by e-cigarette use (p=0.869)

E-cigarette use was most common among the persistent smokers (10.6%), followed by 7.8% among starters, 6.4% among those who relapsed, and 2.1% among those who quit smoking at follow-up (Figure 1). The majority (63.7%) of the persistent smokers who also used e-cigarettes smoked the same number of cigarettes per day at baseline and at follow-up. In comparison, 24.2% smoked a decreased number of cigarettes and 12.1% an increased number of cigarettes per day (Figure 2). These proportions were almost identical among persistent smokers who did not use e-cigarettes. Of the current smokers at baseline, 45.3% (n=1421) had quit tobacco smoking by follow-up, and the estimate was significantly lower among e-cigarette users (14.2%, n=30) than among non-e-cigarette users (47.6%, n=1391, p<0.001).

### Association between respiratory symptoms at baseline and initiation of e-cigarette use

E-cigarette use at follow-up was more common among those with respiratory symptoms at baseline than those without symptoms (Table 2). Moreover, e-cigarette use was more common among current smokers with respiratory symptoms than among smokers without symptoms at baseline.

**Table 2:** E-cigarette use at follow-up in 2016 among those with and without respiratory symptoms at baseline in 2006–2008, and among current smokers with and without respiratory symptoms at baseline^a^

*Respiratory symptoms*	*All at baseline (N=17325)*		*E-cigarette use at follow-up*			*Current smokers at baseline (N=3134)*		*E-cigarette use at follow-up*		
	*n*	*%*	*n/N*	*%*	*p^b^*	*n*	*%*	*n/N*	*%*	*p^b^*
**Longstanding cough**										
No	15533		217/15533	1.4	<0.001	2648		164/2648	6.2	0.003
Yes	1792	10.3	53/1792	3.0		486	15.5	48/486	9.9	
**Sputum production**										
No	14991		198/14991	1.3	<0.001	2458		146/2458	5.9	<0.001
Yes	2334	13.5	72/2334	3.1		676	21.6	66/676	9.8	
**Chronic productive cough**										
No	16633		249/16633	1.5	0.001	2942		191/2942	6.5	0.017
Yes	692	4.0	21/692	3.0		192	6.1	21/192	10.9	
**Recurrent wheeze**										
No	15910		213/15910	1.3	<0.001	2603		159/2603	6.1	0.001
Yes	1415	8.2	57/1415	4.0		531	16.9	53/531	10.0	
**Wheeze last 12 months**										
No	14384		183/14384	1.3	<0.001	2188		129/2188	5.9	0.003
Yes	2941	17.0	87/2941	3.0		946	30.2	83/946	8.8	
**Dyspnea**										
No	16330		234/16330	1.4	<0.001	2841		178/2841	6.3	<0.001
Yes	995	5.7	36/995	3.6		293	9.3	34/293	11.6	
**Any respiratory symptom**										
No	12458		136/12458	1.1	<0.001	1794		92/1794	5.1	<0.001
Yes	4867	28.1	134/4867	2.8		1340	42.8	120/1340	9.0	

a Study design: cohort study. Setting: the counties Norrbotten and Västra Götaland, Sweden. Sample size: n=17325.

b χ^2^ test comparing proportion of e-cigarette use by respiratory symptom.

### Associations between tobacco smoking and e-cigarette use and respiratory symptoms

**Figure 3:**
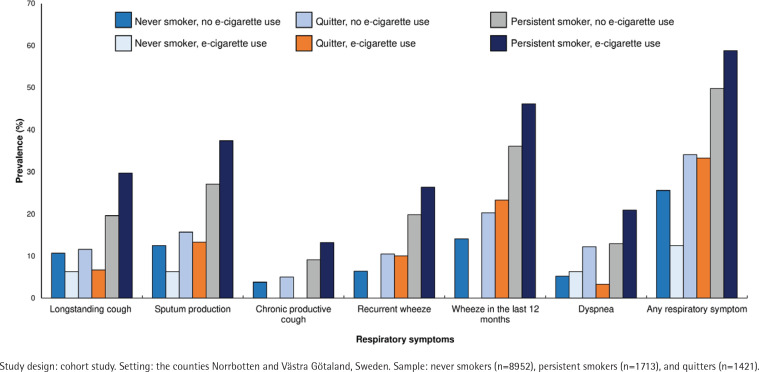
The prevalence (%) of respiratory symptoms among never smokers, quitters and persistent smokers by e-cigarette use; χ^2^ test (and test for linear trend, respectively) comparing prevalence of respiratory symptoms by e-cigarette use and smoking habits showed p<0.001 for each symptom

The prevalence of all respiratory symptoms at follow-up was highest among persistent smokers who used e-cigarettes compared to never smokers, quitters and persistent smokers who did not use e-cigarettes (Figure 3). In general, the prevalence of respiratory symptoms was lower in never smokers than quitters and persistent smokers, and there was no clear pattern between users and non-users of e-cigarettes within each category of smoking status. In a logistic regression analysis, adjusted for sex, age, any respiratory symptoms at baseline and socioeconomic status, persistent smokers without e-cigarette use (OR=2.27; 95% CI: 2.00–2.57) and with e-cigarette use (OR=2.49; 95% CI: 1.78–3.48) had an increased risk for any respiratory symptoms at follow-up, compared to never smokers without e-cigarette use (Supplementary file Table E2).

**Figure 4:**
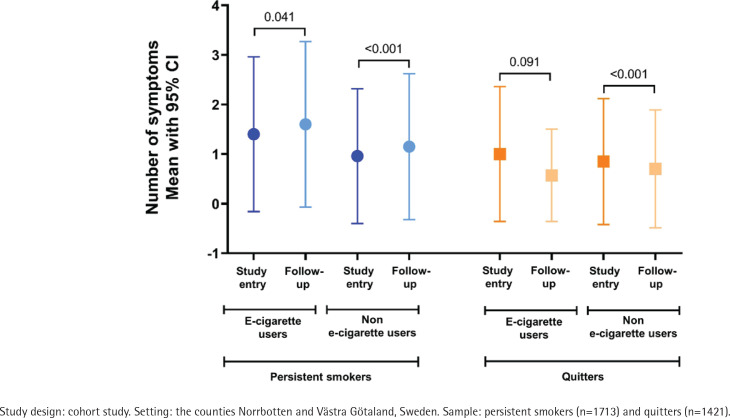
Change in mean number of symptoms among persistent smokers and quitters, among users and nonusers of e-cigarettes, respectively

At baseline, the mean number of respiratory symptoms was 0.55 (SD=1.05) among all, and 0.94 (SD=1.34) among current smokers. Among persistent smokers, there was a significant increase in the mean number of symptoms from baseline to follow-up, both among users and non-users of e-cigarettes (Figure 4). Among quitters who used e-cigarettes, the mean number of symptoms had decreased, but the difference was not statistically significant.

We performed an ordinal regression analysis among current smokers at baseline. We adjusted for sex, age, socioeconomic status and number of symptoms at baseline and evaluated the number of respiratory symptoms at follow-up. Compared with quitters who did not use e-cigarettes (reference category), both persistent smoking with e-cigarette use (OR=2.59; 95% CI: 1.92–3.47) and without e-cigarette use (OR=2.08; 95% CI: 1.79–2.42) was significantly associated with a higher number of symptoms. However, there was no statistically significant difference in the number of symptoms between quitters who did not use e-cigarettes and quitters who were e-cigarette users (OR=0.72; 95% CI: 0.32–1.65).

## DISCUSSION

In this large, prospective population-based study, almost half of the tobacco smokers had quit smoking during the 8–10 years of follow-up. E-cigarette use at follow-up was associated with persistent tobacco smoking, smoking more than 15 cigarettes per day and reporting respiratory symptoms. Furthermore, neither quitting tobacco smoking nor reducing the number of tobacco cigarettes smoked per day was more common among e-cigarette users. Those who were persistent tobacco smokers reported increasing respiratory symptoms during the study period. Respiratory symptoms decreased among those who quit tobacco smoking, but there was no significant association between e-cigarette use and a reduction of respiratory symptoms.

We found that e-cigarette use was more common among those who were tobacco smokers at baseline than among non-smokers or former smokers, similar to other studies among adults^2,10,14^. One explanation could be that smokers who wish to quit initiate e-cigarette use during a transition period with the intention to gradually reduce tobacco cigarettes, thus using both products simultaneously. However, the majority of e-cigarette users had not changed the number of cigarettes smoked per day by follow-up. Moreover, the quit rate of tobacco smoking was considerably lower in e-cigarette users than in non-users of e-cigarettes. The direction of the association is difficult to disentangle. Smokers may initiate e-cigarette use because they have made previous unsuccessful quit attempts and now wish to try something new. Or, smokers who initiate e-cigarette use may struggle to quit because they have initiated e-cigarette use and continue the nicotine addiction. Studies on whether e-cigarette use is associated with a reduction in tobacco smoking also show diverging results. In some studies, e-cigarette users smoked fewer tobacco cigarettes per day^21,22^, while others found that e-cigarette users increased their cigarette consumption^23,24^. Two systematic reviews and meta-analyses have concluded that although randomized controlled trials (RCT) may show higher quit rates among those allocated to e-cigarettes than traditional smoking cessation aids (such as nicotine gum or patches), this association was not seen in population-based studies^11,25^. Taken together, e-cigarette use did not seem to work as a tool for tobacco smoking reduction or cessation on a population level.

We can confirm the hypothesis that tobacco smokers with respiratory symptoms were more likely to initiate e-cigarette use. This may be another indication of smokers having developed respiratory symptoms and wish to quit or reduce their smoking, initiating e-cigarette use as a first step. Furthermore, we found that the prevalence of respiratory symptoms was highest among the persistent smokers who also used e-cigarettes, thus confirming our second hypothesis. Our results are in line with a longitudinal US study, showing that dual use of tobacco and electronic cigarettes increased the risk of respiratory disease more than either product alone^15^. Already among adolescents, it has been shown that the greater the frequency of e-cigarette use, the higher the number of respiratory symptoms^26^. Whether switching from tobacco to e-cigarettes improves respiratory health remains controversial. In one RCT, smokers who were attempting to reduce their consumption of tobacco cigarettes were assigned either e-cigarettes or a non-nicotine-containing cigarette substitute. Although the e-cigarette group showed improvement in some cardiovascular outcomes, no effects on respiratory outcomes were found^27^. In our study, we found that e-cigarette users who had quit tobacco smoking showed decreased symptoms at follow-up, but the difference was not statistically significant. Moreover, there was no association in the number of respiratory symptoms between quitters with and without e-cigarette use. Thus, our third hypothesis could not be confirmed.

Following persistent smokers, the categories with the second and third highest prevalence of e-cigarette use were starters and relapsers, i.e. individuals who were non-smokers or former smokers at baseline and dual users at follow-up. Systematic reviews have shown evidence for an association between e-cigarette use and subsequent tobacco smoking, especially among teenagers, and it has been suggested that former smokers using e-cigarettes had an increased risk of tobacco smoking relapse^28,29^. However, due to the few cases of starters (n=11) and relapsers (n=14) who used e-cigarettes in our study, the association with respiratory symptoms could not be explored. In non-smokers the prevalence of respiratory symptoms was higher among non-users of e-cigarettes than e-cigarette users. One explanation for this finding may be that individuals who already experience respiratory symptoms, for instance, due to asthma, are less likely to initiate any kind of smoking behavior.

Overall, tobacco smoking cessation was common; almost half of the current smokers at baseline had quit at follow-up. One explanation for these high quit rates may be the stricter tobacco law that was introduced in Sweden in 2005, including smoking bans in restaurants and bars. After its introduction, a decrease in smokers in the general population was seen, followed by decreased prevalence of respiratory symptoms and chronic obstructive pulmonary disease^18,30^. Similarly, our study found a beneficial effect of tobacco smoking cessation, probably because respiratory symptoms decreased. This is an important finding on its own for public health; after decades of strengthened tobacco legislation and prevention campaigns, we found both decreased tobacco smoking rates and subsequently improved respiratory health in our random sample of the population.

### Strengths and limitations

The strengths of this study include the longitudinal design, including large, random, representative samples of the population. The same questionnaire was used in OLIN and WSAS, enabling pooling of data from two large Swedish counties that include both urban and rural settings and cities of different sizes and socioeconomic levels. Despite the large sample size, sub-group analyses, for instance, by age group, were not possible due to a relatively low prevalence of e-cigarette use. Another limitation is that we asked for current and not previous e-cigarette use, nor the duration of e-cigarette use. Thus, we could not identify former e-cigarette users or the burden of exposure to e-cigarettes. For instance, smokers at baseline who quit after using e-cigarettes and then quit e-cigarettes, have been classified as quitters without e-cigarettes in our study. The eight to ten years of follow-up time could introduce attrition bias and/or a ‘healthy survivor effect’. Both OLIN and WSAS have performed non-responder analyses and found that neither prevalence estimates of respiratory symptoms nor the associations with risk factors were underestimated^17,19^. However, smokers declined participation to a higher degree than non-smokers, which is why it is unlikely that the associations found in the current study are overestimated.

## CONCLUSIONS

In this population-based study, e-cigarette use was associated with persistent tobacco smoking, smoking more than 15 cigarettes per day, and reporting respiratory symptoms. We did not find any associations between e-cigarette use and quitting smoking or a reduction in number of cigarettes smoked per day. Persistent smokers reported increasing respiratory symptoms, while those who quit tobacco smoking reported decreasing respiratory symptoms, but there was no significant association between e-cigarette use and a reduction of respiratory symptoms.

## Data Availability

The data supporting this research are available from the authors upon reasonable request and after a confidentiality evaluation.
