# Supplementary material for: Electronic cigarette use in relation to changes in smoking status and respiratory symptoms
Source: Tob Induc Dis. 2024 Jan 22;22:10.18332/tid/176949. doi: 10.18332/tid/176949 (PMC10801701; doi:10.18332/tid/176949)
Supplement: Supplementary file 1 [file TID-22-21-s1.pdf]

Table E1. Characteristics of participants at study entry in 2006-2008, by study area and sex.

	All, n=17,325		OLIN, n=7,384		WSAS, n=9,941		Difference by area	Men, n=8,071		Women, n=9,254		Difference by sex
	n	%	n	%	n	%	p-value	n	%	n	%	p-value
**Sex**												
Men	8,071	46.6	3,658	49.5	4,413	44.4						
Women	9,254	53.4	3,726	50.5	5,528	55.6	<0.001					
**Age groups**												
16-19y	435	2.5	0		462	4.4		161	2.0	274	3.0	
20-29y	1,970	11.4	481	6.5	1,489	15.0		798	9.9	1,172	12.7	
30-39y	3,053	17.6	1,298	17.6	1,755	17.7		1,381	17.1	1,672	18.1	
40-49y	3,743	21.6	1,749	23.7	1,994	20.1		1,739	21.5	2,004	21.7	
50-59y	4,421	25.5	2,179	29.5	2,242	22.6		2,140	26.5	2,281	24.6	
60-69y	3,703	21.4	1,677	22.7	2,026	20.4	<0.001	1,852	22.9	1,851	20.0	<0.001
**Smoking habits**												
Non-smoker	9,718	56.1	4,118	55.8	5,600	56.3		4,668	57.8	5,050	54.6	
Former smoker	4,473	25.8	1,925	26.1	2,548	25.6		2,105	26.1	2,368	25.6	
Current smoker	3,134	18.1	1,341	18.2	1,793	18.0	0.744	1,298	16.1	1,836	19.8	<0.001
**Socioeconomic status**												
Professionals and executives	2,270	13.1	1,251	16.9	1,019	10.3		1,160	14.4	1,110	12.0	
Self-employed	239	1.4	88	1.2	151	1.5		165	2.0	74	0.8	
Non-manual employees	5,884	34.0	1,867	25.3	4,017	40.4		2,171	26.9	3,713	40.1	
Manual workers in industry	3,077	17.8	1,590	21.5	1,487	15.0		2,671	33.1	406	4.4	
Manual workers in service	4,176	24.1	2,006	27.2	2,170	21.8		1,245	15.4	2,931	31.7	
Students	1,097	6.3	316	4.3	781	7.9		391	4.8	706	7.6	
Unspecified	582	3.4	266	3.6	316	3.2	<0.001	268	3.3	314	3.4	<0.001

Study design: cohort study; Setting: the counties Norrbotten and Västra Götaland, Sweden; Sample size: n=17,325.

Table E2. The association between smoking habits and e-cigarette use and any respiratory symptoms at follow-up in 2016, analysed by logistic regression and presented as unadjusted and adjusted odds ratios with 95% confidence intervals.

	Unadjusted		Adjusteda		Adjustedb	
	OR	95% CI	OR	95% CI	OR	95% CI
Never smoker, Non e-cigarette user	1.0		1.0		1.0	
Never smoker, e-cigarette user	0.42	(0.09-1.83)	0.50	(0.11-2.40)	0.48	(0.10-2.29)
Quitter, non e-cigarette user	1.50	(1.33-1.69)	1.07	(0.94-1.23)	1.05	(0.92-1.21)
Quitter, e-cigarette user	1.45	(0.68-3.11)	0.84	(0.36-1.94)	0.81	(0.35-1.87)
Persistent smoker, non e-cigarette user	2.88	(2.58-3.22)	2.32	(2.05-2.63)	2.27	(2.00-2.57)
Persistent smoker, e-cigarette user	4.14	(3.07-5.58)	2.60	(1.86-3.64)	2.49	(1.78-3.48)

[a]Adjusted for sex, age and any respiratory symptoms at study entry in 2006-2008.

[b]Adjusted for sex, age, any respiratory symptoms at study entry in 2006-2008, and socio-economic status.

Study design: cohort study; Setting: the counties Norrbotten and Västra Götaland, Sweden; Sample size: n=12,086.

STROBE Statement—checklist of items that should be included in reports of observational studies

	Item No.	Recommendation	Page No.	Relevant text from manuscript
**Title and abstract**	1	(*a*) Indicate the study's design with a commonly used term in the title or the abstract	1	The journal's limit of 90 characters in the title did not allow for this information in the title. However, it is included in the abstract.
		(*b*) Provide in the abstract an informative and balanced summary of what was done and what was found	2	
**Introduction**				

Background/rationale	2	Explain the scientific background and rationale for the investigation being reported	3-4	We have strived to explain the background and rationale for the study.
Objectives	3	State specific objectives, including any prespecified hypotheses	4	We provide both an objective and three hypotheses.
**Methods**				
Study design	4	Present key elements of study design early in the paper	4-5	
Setting	5	Describe the setting, locations, and relevant dates, including periods of recruitment, exposure, follow-up, and data collection	4	Both the geographical setting and relevant dates are presented early in the paper.
Participants	6	(*a*) *Cohort study*—Give the eligibility criteria, and the sources and methods of selection of participants. Describe methods of follow-up<br>*Case-control study*—Give the eligibility criteria, and the sources and methods of case ascertainment and control selection. Give the rationale for the choice of cases and controls<br>*Cross-sectional study*—Give the eligibility criteria, and the sources and methods of selection of participants	a) 4-5	Cohort study: the sources and methods of selection are presented, as well as the follow-up time.
		(*b*) *Cohort study*—For matched studies, give matching criteria and number of exposed and unexposed<br>*Case-control study*—For matched studies, give matching criteria and the number of controls per case		N/A
Variables	7	Clearly define all outcomes, exposures, predictors, potential confounders, and effect modifiers. Give diagnostic criteria, if applicable	5-6	Definitions of exposures and outcomes and the included co-variates are presented.
Data sources/ measurement	8*	For each variable of interest, give sources of data and details of methods of assessment (measurement). Describe comparability of assessment methods if there is more than one group	5	All variables were collected through postal questionnaires.
Bias	9	Describe any efforts to address potential sources of bias	5, 11	The questionnaire has been validated previously. Non-

				responder studies have been performed.
Study size	10	Explain how the study size was arrived at	4-5	Participation and study size is presented.

Continued on next page

Quantitative variables	11	Explain how quantitative variables were handled in the analyses. If applicable, describe which groupings were chosen and why		6-7 This is presented in Statistical analyses
Statistical methods	12	(*a*) Describe all statistical methods, including those used to control for confounding		6-7 This is presented in Statistical analyses
		(*b*) Describe any methods used to examine subgroups and interactions		6-7 This is presented in Statistical analyses
		(*c*) Explain how missing data were addressed		6-7 This is presented in Statistical analyses
		(*d*) *Cohort study*—If applicable, explain how loss to follow-up was addressed<br>*Case-control study*—If applicable, explain how matching of cases and controls was addressed<br>*Cross-sectional study*—If applicable, describe analytical methods taking account of sampling strategy	5,11	Non-responder studies have been performed.
		(*e*) Describe any sensitivity analyses	N/A	
**Results**				
Participants	13*	(a) Report numbers of individuals at each stage of study—eg numbers potentially eligible, examined for eligibility, confirmed eligible, included in the study, completing follow-up, and analysed	4-5	This is presented in Study sampling and procedure
		(b) Give reasons for non-participation at each stage	5	Non-responder studies have been performed and published previously.
		(c) Consider use of a flow diagram	N/A	
Descriptive data	14*	(a) Give characteristics of study participants (eg demographic, clinical, social) and information on exposures and potential confounders	Supplement	This is presented in table e1 in the online supplement

		(b) Indicate number of participants with missing data for each variable of interest	N/A	Those with missing data on the exposure variables were excluded from the sample.
		(c) *Cohort study*—Summarise follow-up time (eg, average and total amount)	4-5	This is presented in Study sampling and procedure
Outcome data	15*	*Cohort study*—Report numbers of outcome events or summary measures over time	7-8, 17	This is presented in the Results text, and in table 1, figure 1, and figure 3.
		Case-control study—Report numbers in each exposure category, or summary measures of exposure		
		Cross-sectional study—Report numbers of outcome events or summary measures		
Main results	16	(*a*) Give unadjusted estimates and, if applicable, confounder-adjusted estimates and their precision (eg, 95% confidence interval). Make clear which confounders were adjusted for and why they were included	7-8	This is presented in the Results, as well as in the table and figures.
		(*b*) Report category boundaries when continuous variables were categorized	N/A	
		(*c*) If relevant, consider translating estimates of relative risk into absolute risk for a meaningful time period	N/A	

Continued on next page

Other analyses	17	Report other analyses done—eg analyses of subgroups and interactions, and sensitivity analyses	N/A	
**Discussion**				
Key results	18	Summarise key results with reference to study objectives	9	The Discussion starts with a summary of main findings
Limitations	19	Discuss limitations of the study, taking into account sources of potential bias or imprecision. Discuss both direction and magnitude of any potential bias	11-12	Limitations and strengths of the study are discussed.
Interpretation	20	Give a cautious overall interpretation of results considering objectives, limitations, multiplicity of analyses, results from similar studies, and other relevant evidence	9-12	This is presented throughout the discussion.
Generalisability	21	Discuss the generalisability (external validity) of the study results	11	
**Other information**				
Funding	22	Give the source of funding and the role of the funders for the present study and, if applicable, for the original study on which the present article is based	12,13	Funders and their role are presented.

*Give information separately for cases and controls in case-control studies and, if applicable, for exposed and unexposed groups in cohort and cross-sectional studies.

Note: An Explanation and Elaboration article discusses each checklist item and gives methodological background and published examples of transparent reporting. The STROBE checklist is best used in conjunction with this article (freely available on the Web sites of PLoS Medicine at http://www.plosmedicine.org/, Annals of Internal Medicine at http://www.annals.org/, and Epidemiology at http://www.epidem.com/). Information on the STROBE Initiative is available at www.strobe-statement.org.

© 2024 Hedman L. et al.
